# Supplementary material for: Incidental vocabulary learning with subtitles in a new language: Orthographic markedness and number of exposures
Source: PLoS One. 2021 Feb 16;16(2):e0246933. doi: 10.1371/journal.pone.0246933 (PMC7919001; doi:10.1371/journal.pone.0246933)
Supplement: S1 Appendix — (DOCX) [file pone.0246933.s001.docx]

**S1 Appendix. Aural texts**

**Video 1**

A continuación mostraremos información de algunos animales que pueblan nuestro planeta y que muestran muy diversas características.

Todo el mundo sabe que el **conejo** se reproduce con suma facilidad.

La carne que devora **el ratón** procede fundamentalmente de la carroña.

Todo en la anatomía del **pájaro** refleja su capacidad para volar ya que en su momento la evolución obró para ello.

También conocidos como quelonios, la **tortuga** es un tipo de reptil caracterizado por el sólido caparazón que protege sus órganos vitales.

La afición al **conejo** hace que se promocione considerablemente su cría.

En cuanto a su clasificación, el **pez** es un animal vertebrado principalmente acuático.

En la naturaleza son presa de otros animales, así que la vida de un **ratón** rara vez excedElos tres meses de duración.

Puesto que es un animal herbívoro, el **conejo** se alimenta fundamentalmente de plantas herbáceas.

El esqueleto del **pájaro** está hecho de huesos huecos pero resistentes.

El periodo de gestación del **conejo** dura aproximadamente un mes y paren entre cuatro a diez ejemplares.

En la naturaleza, la mayoría de las especies de **ratón** son herbívoras, consumen todo tipo de frutas o granos de las plantas.

Las plagas que transmite el **conejo** pueden causar grandes destrozos a la agricultura.

Comparándolo con los vertebrados, el **pez** tiene generalmente un cerebro pequeño en relación al tamaño de su cuerpo.

Normalmente, el **pez** está recubierto de escamas y está dotado de aletas que le permiten un movimiento continuo en medios acuáticos.

La vida en cautividad de un **ratón** puede llegar a los dos años.

El hábitat preferido de la mayoría de razas de **conejo** son las zonas extensas con abundante matorral.

El marcado carácter territorial del **gato** hace que la convivencia con otro felino sea muchas veces un asunto delicado.

Gracias a su sistema digestivo, el **pájaro** puede comer durante el vuelo y digerir el alimento más tarde.

Desafortunadamente la industria peletera utiliza alguna razas de **conejo** con propósitos comerciales.

Un dato curioso es que el **ratón** diferencia los tonos claros y oscuros, pero no pueden distinguir colores.

La forma en que un **pájaro** se reproduce está relacionada con el vuelo, ya que ponen sus huevos y los incuban en un nido, en lugar de llevarlos en su vientre.

Curiosamente, el **ratón** es una de las primeras especies que ha viajado al espacio exterior.

Como mascota, mayoritariamente se prefiere el **conejo** enano, ya que se adapta mejor a la vida en un apartamento.

Es sabido que el **ratón** interesa a los humanos por sus virtudes como animal de laboratorio.

Se sabe que el **pez** realiza la mayor parte del intercambio gaseoso mediante el uso de las branquias.

El más común y conocido es el **ratón** de casa, el segundo mamífero más extendido del planeta tras el ser humano.

Hemos podido ver algunas de las características principales, costumbres y relación con los humanos de distintos animales comunes.

**Video 2**

Se habla mucho acerca de los beneficios de comer fruta, pero conviene ahondar más para saber para qué es conveniente el consumo de las diferentes frutas que encontramos en el mercado.

Ramón Llull fue uno de los primeros en recomendar la **fresa** para diversas afecciones.

En realidad, el **mango** es el nombre de las frutas de varias especies de árboles del género Mangífera.

Una de las propiedades de la **fresa** es que posee una notable riqueza mineral, especialmente de hierro.

Una de las frutas más integradas en nuestra gastronomía es la **uva**.

Hay que tener en cuenta que la **fresa** puede producir reacciones alérgicas por su alto contenido en ácido salicílico.

Lo que conocemos como **mango** comprende muchas variedades de frutas, muchas de ellas obtenidas por injerto.

Una de las propiedades de la **uva** es que es una fruta muy energética, pues contiene abundantes cantidades de hidratos de carbono.

La intensidad del pigmento anaranjado de la pulpa del **melón** está relacionada con la cantidad que tiene de beta-caroteno, de acción antioxidante.

Al igual que otras frutas, la **uva** contiene más de un 80% de agua, que ayuda a aligerar el organismo.

La mejor temporada para comer la **uva** es la que va de agosto a diciembre.

Existen más de 30 variedades de **pera**, de diferentes colores, texturas y sabores.

Se dice que la **uva** es idónea para mejorar el estado de ánimo y reponerse del cansancio.

Se suele recomendar a las embarazadas el consumo de **fresa**, ya que es una fruta con bajo contenido en azúcares.

La melonera, planta que da el **melón**, es tallo rastrero y pertenece a la familia de las Cucurbitáceas.

El nombre botánico de la palabra **fresa** deriva del latín FRAGANS, que significa ‘olorosa’.

Lo que hace de la **uva** una fruta singular es que es una vaya, y como tal es rica en fitonutrientes.

Junto con la frambuesa, la **fresa** es una de las frutas más ricas en hierro.

Aunque acepta cualquier tipo de suelo con un buen drenaje, la **piña** es un cultivo claramente tropical.

El aporte calórico de la **uva** queda compensado por su capacidad depurativa.

En nuestro país el **mango** goza de una gran popularidad: es uno de los frutos tropicales más extendidos.

Las principales zonas de cultivo de la **fresa** en España están en Huelva y en el Maresme Barcelonés.

Las escasas calorías que aporta el **melón** se deben a su contenido moderado de azúcares.

Es sabido que el **mango** aporta una gran cantidad de contenido calórico, por la abundante presencia de hidratos de carbono.

Todo el mundo conoce la capacidad antioxidante que tiene la **uva** en todas sus variedades.

Por su bajo contenido calórico, el **melón** está indicado en dietas de control de peso.

Valencia y Extremadura son grandes productoras de **fresa** dentro de la península.

Hemos podido ver el origen, las características y las bondades de algunas de las frutas que más se consumen en nuestro país.
